# Transcriptomic prediction of breeding values in loblolly pine

**DOI:** 10.1371/journal.pone.0319425

**Published:** 2025-04-23

**Authors:** Adam Festa, Ross W. Whetten

**Affiliations:** Department of Forestry and Environmental Resources, North Carolina State University, Raleigh, North Carolina, United States of America; KGUT: Graduate University of Advanced Technology, Kerman, Iran

## Abstract

Phenotypic variation in forest trees can be partitioned into subsets controlled by genetic variation and by environmental factors, and heritability expressed as the proportion of total phenotypic variation attributed to genetic variation. Applied tree breeding programs can use matrices of relationships, based either on recorded pedigrees in structured breeding populations or on genotypes of molecular genetic markers, to model genetic covariation among related individuals and predict genetic values for individuals for whom no phenotypic measurements are available. This study tests the hypothesis that genetic covariation among individuals of similar genetic value will be reflected in shared patterns of gene expression or shared sequence variation in expressed genes. We collected gene expression data by high-throughput sequencing of RNA isolated from pooled seedlings from parents of known genetic value, and compared alternative approaches to data analysis to test this hypothesis. Selection of specific sets of transcripts increased the predictive power of models over that observed using all transcripts or SNPs. Models using information of both transcript levels and SNP variation showed increased predictive accuracy relative to models using only SNPs or transcript levels. Known pedigree relationships are not required for this approach to modeling genetic variation, so it has potential to allow broader application of genetic covariance modeling to natural populations of forest trees.

## Introduction

Response to selection within breeding programs, commonly referred to as genetic gain, is defined as the change in population mean from one generation to the next due to artificial selection [1]. The amount of time required for one cycle of breeding, testing, and selection is called the generation interval and depends upon a species’ reproductive rate and the time a breeder must wait to make selections. For species with multi-year lifespans, breeders must wait until progeny are old enough to measure commercially-relevant traits in order to obtain accurate breeding value (BV) estimates with high confidence [2,3]. The appropriate age for measurement depends on the trait to be measured, but in some cases age-age correlations of phenotypes are high enough that traits measured at young ages are well-correlated with the same trait at an older age [4]. Accelerating genetic gain by reducing generation intervals or establishing methods to reliably estimate individual BVs sooner could increase the value of any breeding program.

Traditional genetic evaluation in animal and plant breeding relies on phenotypic assessment of individuals and analysis of genetic relationships among relatives. Given the recent increase of genomic resources within many breeding programs, utilizing sequence information to reduce the time estimating BVs has been explored and proven beneficial. One of the first examples of reducing time to estimating BVs through genomic information was genomic selection (GS), which produces dense marker coverage across a genome and takes advantage of associations between markers and phenotypes [5]. Since then, major annual crops such as maize (*Zea mays*), wheat (*Triticum aestivum*), rye (*Secale cereale*), and rice (*Oryza sativa*) have shown improvement within their respective breeding programs through the application of genomic selection [6–9].

In addition, breeding programs for species that have longer generation intervals, such as dairy cattle and horses, have also shown that genomic selection is an efficient method for reducing the time of estimating BVs and thereby decreasing generation intervals [6–8]. Based on the efficiency of genomic selection and the constant reduction in sequencing costs, other ‘omic’ technologies have been explored. Omic methods are aimed at the detection of genes (genomics), mRNA (transcriptomics), proteins (proteomics), and metabolites (metabolomics) in a specific biological sample. These different types of data sets can be used separately or together to infer more about biological processes or as a means of accelerating the estimation of BVs and reducing generation intervals [9–19].

Forest tree breeding programs for species such as loblolly pine (*Pinus taeda*) are more time- consuming than for most crop species, because a single breeding and selection cycle often takes 15 years or more. Additionally, unlike most other breeding programs, tree breeding programs are in their infancy, typically no more than 3 or 4 generations deep with relatively weak pedigrees and connectedness among progeny test datasets [19]. Given this lengthy generation time and the relatively recent inception of tree breeding programs, the utilization of omic information in accelerating generation cycles and assisting in making future selections is an enticing opportunity. Several experiments utilizing omic information for selection in forest trees have been conducted, all of which have focused on using either marker-assisted selection or GS [20–28]. Due to the lengthy generation time of loblolly pine, a complete and robust experiment on utilizing genomic selection within the breeding program will take time to complete. A current experiment is underway to test the efficiency and accuracy of GS within the North Carolina State University (NCSU) Tree Improvement Program (TIP) breeding population using a base population of 2,300 cloned progeny from 53 crosses and is planned to be complete by 2032 [19].

The current selection method for advanced generations of loblolly pine breeding uses family mean and individual-tree phenotypes, where phenotypically superior individuals are selected from top-performing families [22]. Before a selected individual is used in a production population, progeny tests are carried out to confirm the genetic merit of the individual. These progeny tests are grown for four to six years and then measured for phenotypic traits such as volume, height, straightness, and fusiform rust disease incidence (caused by the fungus *Cronartium quercuum f. sp. fusiforme*). BVs of a tree for each trait are then estimated with linear mixed models where phenotype and pedigree data are utilized to help define the genetic covariance among a set of families from a mating design. The BVs determined from progeny tests are analyzed together with a pedigree of known relationships among individuals in the breeding population, using an algorithm to determine which selections should be used in the next breeding cycle to balance performance and amount of relatedness [29]. This requirement to progeny test all candidate selections increases the length of the breeding cycle. One attractive hypothesis is that “omic” selection methods may allow shortening of the breeding cycle, and increasing the rate of genetic gain per year, by allowing earlier selection of elite individuals and confirmation of the genetic value of those individuals.

Genetic differences among families or individuals can be ascribed, at the molecular level, either to differences in gene regulation patterns, or to differences in the structure of the encoded gene products [30]. It is reasonable to propose that genetic variation in family mean phenotypes can be accounted for partly by differences in gene sequence and partly by differences in gene regulation. Published work in maize has reported accurate predictions of genetic differences among F1 hybrid progeny field performance based on pair- wise comparisons of gene expression levels among seedlings of the inbred parent lines [31]. Those authors identified a set of genes differentially expressed in at least one pair-wise comparison among the seedlings of different parental lines, then used gene expression data obtained from microarray experiments to create covariance matrices, either using the quantitative differences in expression or simple binary comparisons [31,32]. Utilizing a cross-validation approach with statistical models trained with a subset of the data, they confirmed that similarities in patterns of gene expression are correlated with similarities in field performance. We hypothesized that RNA-seq analysis of seedlings of loblolly pine might provide the basis for similar transcriptome-based prediction of genetic value. This hypothesis does not propose that juvenile patterns of gene expression determine mature traits, either in maize or in loblolly pine - rather, the hypothesis is that patterns of juvenile gene expression are consistently correlated with patterns of mature gene expression, which in turn determine the phenotypes of genetic entries at the mature stage.

Loblolly pine does not have inbred lines or genetically-uniform hybrid F1 families; instead, the genetic entries used for deployment are either open-pollinated (OP) families of seedlings with a common seed parent, or control-pollinated (CP) families with both pollen and seed parents in common. An approach to transcriptomic prediction appropriate to loblolly pine would be to use family-mean levels of gene expression values, because different loblolly pine families are known to have different genetic values for traits of interest. The most cost-effective method to estimate gene expression levels is by sequencing DNA copies of messenger RNA; this has been found to include less systematic bias when compared to microarray-based expression profiling due to the lack of background hybridization and has the additional benefit of identifying novel mRNA isoforms [33,34]. Obtaining gene expression estimates from RNA-seq typically involves first fragmenting long RNA sequences and then converting the RNA fragments into short complementary DNA (cDNA) fragments. These fragments are then sequenced on a high- throughput instrument and aligned back to a transcriptome or genome to quantify the number of times a given gene or isoform has been detected. This initial quantification of gene expression estimates is systematically biased by many things, including the gene length, the batch that samples were sequenced in, and the lane or index used during the sequencing process [35]. To correct these systematic biases, transcript counts are typically normalized using various statistical approaches and then further used to address hypotheses in a biological context. However, there is still no “gold standard” for normalizing the expression of RNA-seq reads accurately.

Family-mean gene expression values obtained from RNA-seq may serve as a basis for estimation of the genetic covariance in gene regulation patterns. The DNA sequencing approach also provides information on DNA sequence variation such as single-nucleotide polymorphisms (SNPs) that can potentially reveal the genetic covariance among families with respect to gene structure. Utilizing these covariance structures for the prediction of parental BV’s, with or without selection of specific subsets of SNPs or transcripts to improve the signal-to-noise ratio, may provide predictive accuracy comparable to that obtained from family-based relationship models. Recent software tools allow for the incorporation of multiple covariance matrices, which may be used to evaluate the predictive power of these types of genomic information. We chose to use RNA-seq to measure both transcript abundance and assess sequence variation in coding regions of expressed genes, rather than adopting separate methods to measure gene expression (e.g. microarrays) and DNA sequence variation (e.g. exome-capture sequencing). Exome-capture sequencing has been applied to detect sequence variation in coding sequences in loblolly pine [36,37], but this approach is confounded by the presence of processed pseudogenes in conifer genomes [37], and also does not provide any measure of which genes are actually expressed or at what levels. No available genomic technology could provide genome-wide measurements of both gene expression levels and gene sequence variation from a single assay, but RNA-seq is a reasonable option to use for an initial test of the hypothesis that covariation in gene expression levels and gene sequence variation will be associated with phenotypic variation among families in loblolly pine.

The objectives of this study were to (1) collect data on gene expression and SNP variation in expressed genes from a set of families of known breeding value, and assess the reproducibility of gene expression estimates obtained through sequencing replicate samples of seedlings from open-pollinated (OP), pollen-mix (PMX), or control-pollinated (CP) families, (2) compare methods for utilizing family-mean estimates of gene expression levels or SNP genotypes to predict breeding values in cross-validation studies, (3) compare the predictive accuracy of models utilizing all coding sequence SNP variation or gene expression level variation with models using filtered subsets of SNP genotypes or gene expression levels, and to compare the predictive accuracy of models using either SNP genotypes or gene expression levels with models using both data types, and (4) compare the accuracy of transcriptome-based prediction with previous GS studies in loblolly pine and other conifer species. The overall goal of this study is to test methods for transcriptome-based prediction of breeding values to determine which may be suitable for use in applied tree breeding programs.

## Materials and methods

### Experimental design

#### Plant material.

Seedlings from 93 families (88 unique families) were planted in three separate batches, 46 families in batch one, 25 families in batch two, and 22 families in batch three over three different years. Families grown were produced from selections of the NC State University Cooperative Tree Improvement Program (NCSU-TIP). Across the first two batches, a total of five families were repeated. Seed used for families were obtained from a $-20^{\circ }$C freezer stored at NCSU-TIP. The storage of seed is assumed to be the same for all families; however, the age of the seed differed. All families were stratified, sown, grown, and processed using the same conditions.

#### Seedling growth.

Stratification of seed was done by placing 150 seeds from each family in a plastic bag of water containing 1% hydrogen peroxide. They were stored at 4^∘^C for 24 hours before draining and placed in moist bags at 4^∘^C for 40 days. After stratification, seeds from each family were rinsed and planted into a subsection of a tray in a greenhouse located at NCSU in Raleigh, NC, USA. Trays were filled halfway with medium-coarse vermiculite as the soil medium. Prior to sowing seed, trays were lined with newspaper to prevent any leakage of vermiculite. After sowing a family on a subsection of the tray, a thin layer of vermiculite was spread on top of the tray to cover the seed after watering. The greenhouse was set to 24^∘^C for all batches, and water was applied to seedlings three times a day.

#### Sample collection.

Families in the three separate batches were planted in independent years: batch one (September 2015), batch two (June 2016), and batch three (May 2018). Seedlings were allowed to grow for eight weeks after planting until a ‘rolling’ sample collection began. All samples within a given batch were harvested on a single day between 10 a.m.–1 p.m., to minimize RNA expression differences due to time of day. Prior to collection, sample tubes for each family were labeled, where a single tube represents a single biological replicate of a given family. The number of biological replicates sampled depended on the number of seedlings germinated. If  > 90 seedlings germinated for a family, three biological replicates each containing 30 seedlings were collected. If  < 90 seedlings germinated, the biological replicates each consisted of 15 seedlings, and the number of replicates depended on the number of germinated seedlings. No other variation in the number of seedlings per biological replicate was allowed; for example, if 55 seedlings grew, only three replicates were taken to ensure comparable samples across replicates. Families that produced fewer than 15 seedlings were not collected. Over the first two batches, 65 out of the original 71 had enough seedlings to collect samples. The first year contained 144 biological replicates from 46 families, and the second year contained 61 replicates from 19 families. All 22 families in batch three had enough samples to be collected and contained 66 biological replicates. A list of families sampled from each year and the numbers of biological replicates are represented in S1 File. All biological replicates for an individual family were collected on the same day. Each replicate was collected using the following method:

- 30 or 15 seedlings from the tray subsection were gently removed from the planting mixture to retain roots and rinsed in water to remove vermiculite and debris
- A funnel prechilled on dry ice was then used to transfer ground seedlings to the pre- labeled prechilled tube sitting on dry ice.
- Tubes were stored on dry ice until transferred to a -80^∘^C freezer.
- Between processing each family, the mortar, pestle, and funnel were wiped with 100% ethanol to prevent cross-contamination among families.
- Before RNA extraction, all samples were ground using a coffee grinder, adding dry ice pellets to keep the tissue frozen. Each family was processed one at a time, and the coffee grinder was cleaned with 100% ethanol in between each family to minimize cross-contamination.

#### RNA extraction and sequencing.

Two independent RNA extractions were completed for each biological replicate, and the resulting RNA was pooled into one tube representing a single biological replicate. RNA extractions were done using a 96-well plate protocol modified from published methods for high throughput DNA extraction [38].

For batch one and two, each pooled biological replicate was partitioned into three technical replicates and sequenced using multiplexing across multiple lanes. Biological replicates were randomly assigned to one of 24 sequencing adapter index sequences, and technical replicates were randomly allocated to lanes. Each sequencing lane contained technical replicates with 24 different index sequences. The experimental design for each of the first two batches can be found in S1 File. Library creation, multiplexing, and Illumina HighSeq 2500 single- end sequencing were carried out by the Genomic Sciences Laboratory (GSL) at North Carolina State University in Raleigh, NC, USA. Batch three families were not partitioned into technical replicates; instead, biological replicates were sequenced directly. Library creation, multiplexing, and Illumina NovaSeq paired-end sequencing were carried out by Novogene at Davis, CA, USA.

Expression data received from the GSL and Novogene were processed using bbduk [39], clipping the first (left) 10 bases, filtering adapters, and quality trimming to a Phred20 score. Only the first read of each pair in batch three was used to make all batches as similar as possible. Trimmed reads were used to estimate gene expression levels on a biological replicate level using Sailfish [40] using a loblolly pine transcriptome produced at Indiana University by Don Gilbert. The complete transcriptome assembly was downloaded from http://arthropods.eugenes.org/EvidentialGene/plants/pine/, and filtered to retain only those assembled transcripts that fall into the main, maina2, noclass, and noclassa2 groups and are designated “complete” with cds greater than or equal to 100 aas, supplemented with those in the same groups that are designated “partial” if they are greater than or equal to 100 aa and have a CDD or Arabidopsis BLAST hit. The filtered transcriptome assembly is available at (DOI: 10.5281/zenodo.13376646); this is henceforth referred to as the 78k transcriptome because it contains 78,213 putative transcript contigs.

#### Evaluation of expression similarity.

Reproducibility of RNA-seq read counts per transcript across biological replicates and family mean values used spearman-rank correlation analyses. The Spearman correlation coefficient ($r_{s}$) is defined as the Pearson correlation coefficient between a set of ranked variables. For *n* number of transcripts, the counts within each pair of individuals $X_{i}$ and $Y_{i}$ are converted to ranks, $rgX_{i}$ and $rgY_{i}$ respectively. The Spearman correlation coefficient between the two individuals is then estimated as:


$$\begin{array}{ccc} r_{s}=p_{rg_{X},rg_{Y}}=\frac{cov(rg_{X},rg_{Y})}{\sigma_{rg_{X}}\sigma_{rg_{Y}}} & & \end{array}$$ (1)

where *p* is the Pearson correlation coefficient applied to the ranked variables, $cov(rg_{X},rg_{Y})$ is the covariance of ranked variables, $\sigma_{rg_{X}}$ and $\sigma_{rg_{Y}}$ are standard deviations of the ranked variables. Relative to the Pearson correlation, rank-correlation is not as prone to being impacted by outliers, which reduces the impact of having multiple low- or highly-expressed transcripts on the resulting correlation coefficient. All correlation estimates were constructed using log-base-2 transformed Sailfish counts of all 78,213 putative transcripts.

#### SNP identification.

Candidate SNPs were identified by aligning filtered and trimmed fastq files of all replicates across the three batches to the 78K loblolly pine transcriptome one at a time using bowtie2, producing individual SAM output files [41]. Samtools was used to convert SAM files to BAM files and sort them [42]. Picard was used to merge BAM files of biological replicates into a single BAM file representing one family replicate [43]. Bamaddrg was used to add read groups to files [44]. SNP calling was done using freebayes with settings to ignore indels, multi- nucleotide polymorphisms, complex events, and assuming diploidy of the samples [45]. SNPs reported in the VCF output from freebayes were further filtered using VCFtools to retain candidate SNPs with QUAL values  >  30, minor allele frequency (MAF)  >  0.05, and no missing data. The filtered VCF file was processed with VCFtools to recode SNP genotypes into allele content: 0, 1, or 2 copies of the alternate allele [46].

To evaluate the initial relatedness of families, the allele content matrix was converted to a standard genomic relationship matrix *G* using the first method of VanRaden.[47]


$$\begin{array}{ccc} G=\frac{MM^{\prime }}{2\overset{m}{\underset{k=1}{\sum }}p_{k}(1-p_{k})} & & \end{array}$$ (2)

where *M* was the minor allele frequency (MAF) adjusted genotype matrix with elements $\left(0-2p_{j}\right)$, $\left(1-2p_{j}\right)$, and $\left(2-2p_{j}\right)$ representing genotypes AA, AB, and BB, respectively; $p_{j}$ was the MAF of the $j^{th}$ SNP. The construction of the *G* matrix was done using the AGHmatrix package, and a histogram of the relationship estimates between the training and test families was plotted in R for each set of predictions [48,49].

### Prediction of parental breeding values

Family empirical breeding values for volume were obtained from the NCSU TIP internal database. Breeding values for these parents were calculated from a wide range of testing sites and different trials. The breeding values estimated from progeny field trials are used as “phenotypes” in our cross-validation studies, but it is important to note that these breeding values are much better estimates of the true genetic value of each family than are individual-tree phenotypic measurements. All prediction strategies used features from either SNPs or transcripts on the family-mean level and were conducted in R using the packages OmicKriging and glmnet [50,51].

The OmicKriging model implements a statistical method for optimal or best unbiased linear prediction by using a composite similarity matrix to make predictions based on the known relationships among other individuals [50]. The prediction of a test individual in OmicKriging is computed as the weighted average of the phenotype of individuals in the training set.


$$\begin{array}{ccc} Prediction(Y_{new})=\omega_{1}Y_{1}+\omega_{2}Y_{2}+\cdots +\omega_{n}Y_{n} & & \end{array}$$ (3)

where weights $\omega_{i}$ are defined as a function of all *n* ( *n* + 1 ) ∕ 2 pairs of similarity measures, and *Y* corresponds to the individual phenotype. The weights prescribed by OmicKriging are defined as


$$\begin{array}{ccc} \omega =\Sigma^{-1}\rho & & \end{array}$$ (4)

where *ρ* is a similarity vector between any test individual and the remaining training individuals and *Σ* is a similarity matrix of individuals within the training set. A single composite similarity matrix was used for prediction so that


$$\begin{array}{ccc} \Sigma =\theta_{1}S_{1}+(1-\theta_{1})I & & \end{array}$$ (5)

where the weights *θ* are supplied to each similarity matrix ($S_{i}$) and *I* is the identity matrix used to represent an environmental component independent across individuals. All predictions with OmicKriging were conducted using a Pearson correlation matrix with a weighted value of 1.

In most genomic resources, highly correlated features (i.e. SNP genotypes or gene expression levels) are common when *n* < < *p* (where *n* is the number of samples and *p* is the number of features), leading to a need to reduce the extent of collinearity by selecting a subset of features for predictive modeling. To contrast OmicKriging with a model that intrinsically applies feature selection, we evaluated elastic net (EN) with the R package glmnet [51]. EN is referred to as a compromise between ridge and LASSO regression penalties. Ridge regression has the drawback of not performing feature selection and shrinks coefficients of correlated variables toward one another. At the same time, LASSO can only choose at most *n* variables and typically selects the strongest of highly correlated variables. Given that we expect transcript and SNP data to have a significant amount of highly correlated features and that most features are unrelated to phenotype, utilizing EN allows us to enable feature selection while not limiting ourselves to only *n* variables.

The glmnet model with EN contains two tuning parameters, alpha (*α*) and lambda (*λ*), used to tune the penalty term


$$\begin{array}{ccc} \lambda \left(\frac{1}{2}(1-\alpha )\beta^{2}+\alpha |\beta |\right) & & \end{array}$$ (6)

of the equation [51]:


$$\begin{array}{ccc} {\left(y-\beta_{0}-X^{T}\beta \right)}^{2}+\lambda \left(\frac{1}{2}(1-\alpha )\beta^{2}+\alpha |\beta |\right) & & \end{array}$$ (7)

The parameter *α* controls the type of shrinkage, and parameter *λ* controls the amount of shrinkage. EN prediction was performed with the caret package in R.[52] In order to choose the tuning parameters, the training set was bootstrapped 25 times using a grid search with an alpha sequence of 0.1 to 0.9 by 0.1 and a lambda of 1, 0.1, 0.01, and 0.001.[52] The best-tuned model on the training set, evaluated by the root mean squared error (RMSE), was used for prediction on the test set.

#### Creation of test and training sets.

To assess the ability to predict family breeding values of seedlings grown across multiple batches, batch one was used as the training population to predict batch two and three as the test population. To evaluate if combining batches impacted prediction, individual predictions of batch two and three were conducted using batch one plus the other batch as training to predict the families in the left-out batch. In all cases, families that overlapped between batch one and two were used in the training set and not in the prediction set. We chose to keep the individual batches of RNA-seq data separate, as they would be if the method were used in a breeding program.

#### Prediction using transcripts.

To make predictions on the family level, we calculated the mean of read counts aligned to each putative transcript in the reference transcriptome across biological replicates corresponding to a single family. The family-mean read counts were then added to one (to eliminate zero values) and log-transformed. To remove transcripts with low expression levels, we compared removing transcripts that had less than a mean of two, or a mean of three, family counts on the log2 scale. The resulting set of transcripts which passed this threshold were used as a baseline prediction in OmicKriging and glmnet (this set of predictions is referred to as ALL in result tables). A oneway ANOVA was performed to assess the batch effect on family-mean log2 transformed counts, to identify transcripts that show batch effects. The five families that were present in both batch one and two were removed prior to conducting ANOVA. There are too few families in common to allow modeling and correcting the batch effect within the ANOVA model, because most families occur only in only one batch, and the family effect is therefore confounded with the batch effect. The p-values from ANOVA were adjusted using the lfdr function from the R package qvalue [53], and any transcript with a significant effect of batch on read counts (adjusted P  <  0.05) was removed. Prediction with this set is referred to as setA in the results. To test if eliminating correlated transcripts improved prediction, a correlation matrix of the batch effect filtered set of transcripts was evaluated with the findCorrelation function in the R package caret. Transcripts that explained more than 25% of the variation in another transcript were removed, and the resulting set was then used for prediction with OmicKriging and glmnet. In the results, prediction with removal of correlated features is referred to as setB. The Spearman correlation of transcripts in each training set against the phenotypes was performed to assess if features could be subset further. Transcripts that had an absolute correlation greater than 0.05 with phenotype were then included for prediction; these predictions are referred to as setC.

#### Prediction using SNPs.

To remove any SNPs affected by batch, a one-way ANOVA was performed to assess the effect of batch on the allele counts in the family SNP genotype matrix. Any SNP in which the effect of batch on genotype resulted in a p-value less than 0.05 was removed, with no correction for multiple testing; this set of SNPs is referred to as ALL in the results table. The relative variance was identified for each SNP by dividing the variance of SNP genotypes by the mean genotype for each locus. All SNP loci with values greater than 1 using this ratio were subset and used for predictive modeling, referred to as setA. Pairs of SNP loci with correlated genotypes were identified using the findCorrelation function in R and one member of each pair was dropped; the remaining SNPs are referred to as setB. Additional filtering was explored by generating anovaScores using training data and phenotypes with the R package caret. SNPs significantly associated with phenotypic variation in the training population (P  <  0.05) were used for predictive modeling; these results are referred to as setC.

#### Feature enrichment analysis.

The gProfiler:gOST webserver (https://biit.cs.ut.ee/gprofiler/gost) [54] was used to evaluate possible enrichment of specific Gene Ontology (GO) categories in four sets of pine transcripts: setB and the three different setC version produced from training on batch one alone, on batches one and two, or on batches one and three. This webserver uses Arabidopsis genome annotation as the source of GO terms for each gene, so the first step was to carry out a BLASTX search of Arabidopsis using the pine 78k transcriptome sequences to identify the top BLASTX hit for each transcript in the four sets to be analyzed. A unique list of all Arabidopsis gene products with hits from pine transcript queries was also produced, as this is needed to provide the base list of GO terms against which the terms of each of the four transcript sets was compared. The gene identifier of the top Arabidopsis subject for each pine transcript query in each set was recovered and compiled into a multi-query list, which was then uploaded to the gProfiler:gOST webserver for analysis.

#### Measure of accuracy.

Predicted and observed breeding values were evaluated using simple linear regression and the r and p-value estimates between the two values to quantify prediction accuracy for a given model. ANOVA was used to compare models based on SNPs alone or transcript levels alone with a model using both transcripts and SNPs to determine if the combined model has higher accuracy than either of the two simple models. The mean of prediction values within a given model was taken when evaluating the combination of transcripts and SNPs.

## Results

### Illumina sequencing and sequence data analysis

The samples from the first two batches yielded 3,639,060,450 126-nt reads, and samples from the third batch produced 1,444,563,627 150-nt reads. After trimming sequence adapters and low- quality regions, 2,605,756,024 reads (71.6%) remained in batch one/two and 1,237,931,198 reads (85.7%) in batch three. The average length of trimmed reads was 112 base pairs within batch one and two, and the average base quality was 37.1 using the logarithmic Phred quality score first described by Ewing et al [55]. The average length of trimmed reads for batch three was 140 base pairs, and the average base quality was 36.1.

Across all three batches, total read counts per biological replicate prior to applying any transformation ranged from 10.56 million to 39.24 million, with a mean of 20.76 million reads. For the 78,213 transcript contigs in the reference transcriptome assembly, read counts summed across all 205 biological replicates ranged from a minimum of 0 reads to a maximum of 294.96 million, with a mean of 67,688 reads. Out of the total 78,213 putative transcripts, a total of 40,942 (52.3%) transcripts passed the threshold of having a mean of at least two counts and 34,942 (44.7%) transcripts passed the threshold of having a mean of at least three on the log2 scale across all families. The log2 mean count across all putative transcripts within each of the 78 families ranged from 5.646 to 7.583 and standard deviations ranged from 2.408 to 2.759.

Approximately 3.92 million putative SNPs were initially identified from the merged bam files using freebayes. Filtering the candidate SNPs for those with a minor allele frequency no less than 0.05, a minimum Phred-scaled quality score of 30, and no missing data resulted in 214,449 candidate SNPs. The results from on-way ANOVA testing for batch effects on SNP genotypes resulted in the removal of 22,785 SNPs, leaving 191,655 SNPs remaining in the ALL set.

Out of the 205 biological replicates, 18 biological replicates were removed, 15 biological replicates showed incorrect allele sharing relationships in a SNP relationship matrix, and three biological replicates corresponding to a single family were missing an accurate phenotype. This left 187 biological replicates comprised solely of NCSU TIP Coastal selections for analysis. Due to consolidation of some pollen mix and open-pollinated families, as well as the number of families that had enough germinated seedlings to prepare sequencing libraries, 78 families were used in prediction: 45 in batch one, 11 in batch two, of which five are present in both batch one and two, and 22 in batch three.

### Relationships of individuals using transcript or SNP data

**Fig 1:**
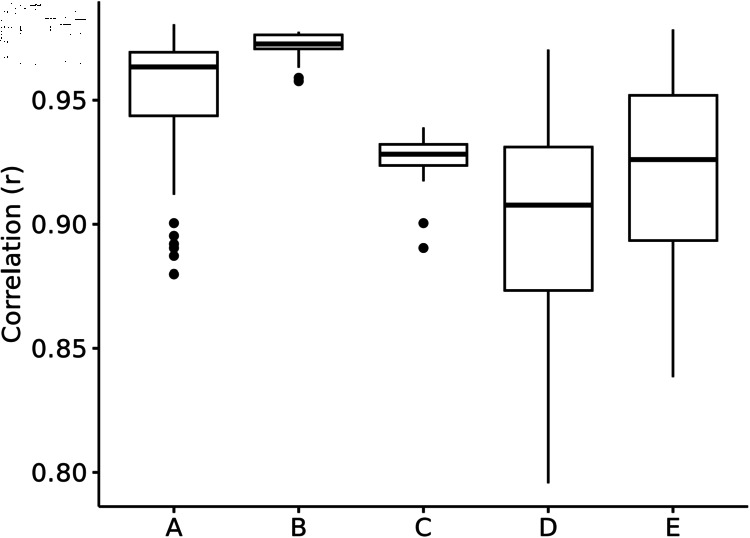
Spearman correlation of log2-transformed gene expression estimates. A: Open-pollinated biological replicates within family (N  =  329). B: Control-pollinated biological replicates within family (N  =  23). C: Biological replicates across batches (N  =  40). D: Biological replicates among family (N  =  32045). E: Family-mean gene expression counts (N  =  3003).

**Fig 2:**
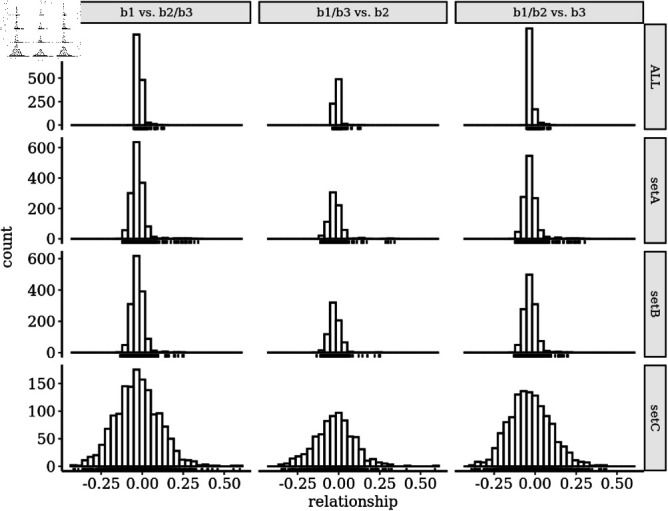
Estimated VanRaden relationships between training and test populations. Three different divisions of batches one, two, and three into training versus test populations are shown from left to right, and four different sets of SNPs at increasing levels of filtration are shown from top to bottom.

To address the consistency in gene expression estimates among open-pollinated or control- pollinated families, correlations among biological replicates within each respective family were evaluated, ranging from 0.87 to 0.97 and 0.95 to 0.97 respectively (Fig 1). The similarity of gene expression estimates across batches grown in different years ranged from 0.89 to 0.94 (Fig 1). Correlation of biological replicates among families ranged from 0.80 to 0.96 (Fig 1). The 3003 comparisons of family pairwise correlations showed overall gene expression patterns to be highly similar across families, with pairwise Spearman correlations ranging from 0.85 to 0.98 (Fig 1). Relationships among training and test families using the 191,664 SNP genomic relationship matrix (ALL in results) showed minimal relationships, as shown in (Fig 2). The additional filtering of SNPs by relative variance, removing correlated features, and conducting an ANOVA to test for significant associations between SNP genotypes in training families and the family phenotype caused an increase in variation of estimated relationships, both in the positive and negative directions. This increased variation seems more likely to be due to violating assumptions in the VanRaden model, rather than to any causal association of the SNPs with phenotype, but resolving these alternatives is beyond the scope of this study.

### Prediction of breeding values using a single batch

When predicting with all data, using a covariance matrix based on SNP genotypes had higher predictive accuracy (assessed as the correlation coefficient of predicted versus observed breeding values) than using covariances based on transcript abundance or on a combination of SNP genotypes and transcript abundance, using either OmicKriging (P  =  3.75e-3) or EN (P  =  1.42e-3) (Table 1). We compared prediction accuracies from data prepared using two different thresholds of removing transcripts which had low expression (mean of two counts within family versus a mean of three counts on the log-2 scale) and concluded that predictions were similar enough with no residual differences; therefore, remaining analyses were conducted using a threshold of eight counts, or a log-2 transformed family mean count of three (S1 File).

**Table 1:** Correlations of predicted and observed breeding values—batches two and three.

	OmicKriging			ElasticNet		
*Filter*	SNPs	Transcripts	Combined	SNPs	Transcripts	Combined
*ALL*	**0.49** ^*^	0.18	0.36^*B^	**0.53** ^*^	0.27	0.42^*B^
*setA*	0.51^*^	0.51^*^	**0.58** ^*^	0.61^*^	0.52^*^	**0.64** ^*B^
*setB*	0.57^*^	0.57^*^	**0.65** ^*AB^	0.67^*^	0.64^*^	**0.72** ^*AB^
*setC*	0.69^*^	0.54^*^	**0.70** ^*B^	0.71^*^	0.62^*^	**0.74** ^*AB^

Correlation (*r*) values of breeding values predicted by OmicKriging or ElasticNet software vs. observed breeding values of families in batches two and three (N  =  33) using a model trained on batch one (N  =  45). Superscript * indicates a correlation p-value less than 0.05, superscript A indicates a combined model ANOVA fit significantly better than SNPs alone, and superscript B indicates a combined model ANOVA fit significantly better than Transcripts alone. Bold values highlight the strongest correlation within each software environment.

Application of a relative variance threshold  >  1 to the data removed 173,105 SNPs with 18,539 remaining for prediction (setA); (S1 File). Compared to using all SNPs, the resulting set improved accuracy using the EN model (P  =  1.63e-4); however, use of this subset of SNPs in OmicKriging had relatively the same correlation which was significant (P  =  2.63e-3) (Table 1).

Applying one-way ANOVA to transcripts and batch with lfdr  <  0.05 resulted in retaining 1,747 transcripts out of the original 34,942 (setA) and the correlation of predicted versus true breeding values were significant in both OmicKriging (P  =  2.27e-3) and EN (P  =  1.78e-3) (Table 1). The highest correlation among setA predictions was observed by taking the mean of predictions from transcripts and SNPs, for both OmicKriging (P  =  4.34e-4) and EN (P  =  5.08e-5) (Table 1).

Removing correlated features from the setA SNPs and transcripts resulted in retaining 5,174 out of 18,539 SNPs and 890 out of 1,747 transcripts (setB). Similar to setA predictions, combining SNP and transcript EN predictions resulted in the highest correlation across all of setB (P  =  1.88e-6) (Table 1). The ANOVA analysis showed modeling the combination of SNP and transcript predictions was significantly better than using either SNPs or transcripts by themselves for both OmicKriging and EN.

**Fig 3:**
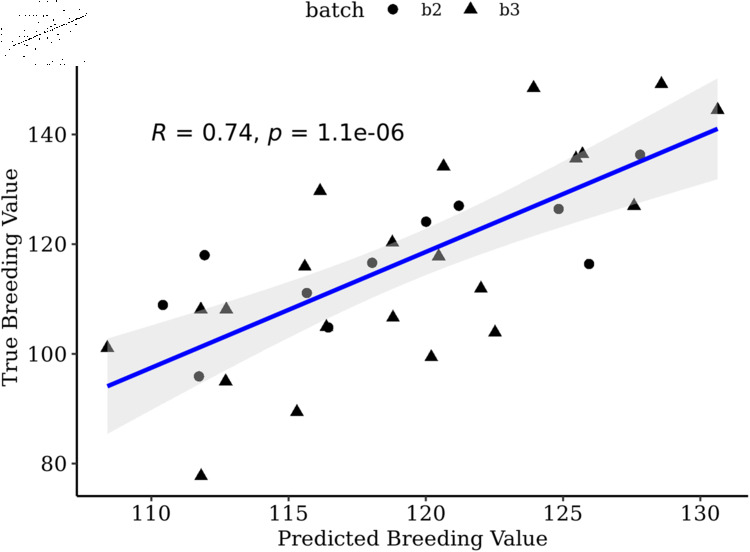
Predicted versus observed breeding values for batch two and three. Predicted versus true breeding values of batch two (N  =  11) and batch three (N  =  22) families using setC combined predictions with EN. Training set included only batch one (N  =  45).

The one-way ANOVA estimates comparing the 5,174 SNPs of setB to volume breeding values did not produce adjusted p-values less than P  =  0.05; therefore, we filtered on unadjusted pvalues, which resulted in keeping a total of 276 SNPs (setC). Selection of transcripts correlated greater than *r*  =  0.05 with phenotypes retained 668 transcripts (setC). Similar to the previous sets, combining the two data types performed the best in both OmicKriging (P  =  6.06e-6) and EN (P  =  1.06e-6) (Table 1). Furthermore, the ANOVA analysis showed the combination of SNP and transcript predictions from EN was significantly better than using SNPs alone (P  =  3.19e-2) or transcripts alone (P  =  1.00e-3). However, when using OmicKriging the combined predictions were significantly better than only transcripts (P  =  4.29e-4) and not SNPs (P  =  7.38e-2). Overall, the highest correlation was observed combining the two data types using setC with EN (Fig 3).

### Prediction of breeding values using two batches for training

#### Batch two predictions.

**Fig 4:**
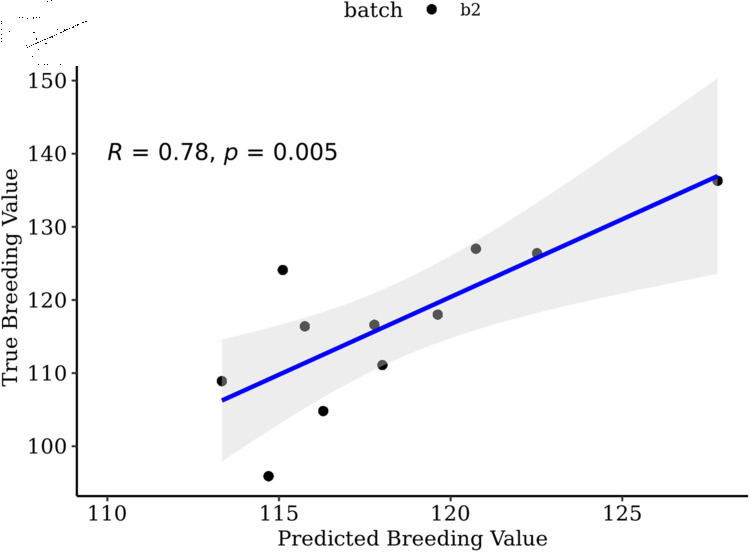
Predicted versus observed breeding values for batch two. Predicted versus true breeding values of batch two (N  =  11) families using ALL SNPs with EN. Training set included batch one and batch three (N  =  67).

Prediction of batch two (N  =  11) when training on batch one and three (N  =  67) resulted in the highest accuracy using all SNPs for both OmicKriging (P  =  5.08e-3) and EN (P  =  5.03e-3) (Table 2). Correlations were nearly identical between the two statistical methods, EN had a slightly lower p-value (Fig 4).

**Table 2:** Correlations of predicted and observed breeding values—batch two.

	OmicKriging			ElasticNet		
*Filter*	SNPs	Transcripts	Combined	SNPs	Transcripts	Combined
*ALL*	**0.78** ^*^	0.23	0.54^B^	**0.78** ^*^	0.60	0.75^*B^
*setA*	**0.68** ^*^	0.50	0.63^*^	**0.64** ^*^	0.41	0.56
*setB*	**0.58**	0.47	0.56	**0.53**	0.33	0.46
*setC*	0.43	0.44	**0.49**	**0.52**	0.36	0.46

Correlation (*r*) values of breeding values predicted by OmicKriging or ElasticNet software vs. observed breeding values of families in batch two (N  =  11), using a model trained on batches one and three (N  =  67). Superscript * indicates a correlation p-value less than 0.05, superscript B indicates a combined model ANOVA fit significantly better than Transcripts alone. Bold values highlight the strongest correlation within each software environment.

Utilizing transcripts, prediction accuracies in EN were moderate, however none of the p-values of the correlations were significant, ranging from P  =  0.31 with setB to P  =  0.051 using all data. Similarly, none of the p-values of the OmicKriging correlations were significant, ranging from P  =  0.49 using all data to P  =  0.16 using setA (Table 2).

One-way ANOVA tests of association of the 5,174 SNPs with training population breeding values resulted in 322 SNPs, and selection of transcripts correlated greater than *r*  =  0.05 with phenotypes retained 613 transcripts (setC). In general, these sets typically performed among the worst within each data type and model (Table 2).

#### Batch three predictions.

Across both OmicKriging and EN, the prediction of batch three families (N  =  22) when training on batch one and two (N  =  56), generally resulted in the highest accuracies when combining SNPs and transcripts within each filter type (Table 3). In the two cases where using only one data type produced the highest correlation, the combination of transcripts and SNP predictions were nearly equivalent (Table 3).

**Table 3:** Correlations of predicted and observed breeding values—batch three.

	OmicKriging			ElasticNet		
***Filter***	**SNPs**	**Transcripts**	**Combined**	**SNPs**	**Transcripts**	**Combined**
*ALL*	0.44^*^	0.43^*^	**0.45** ^*^	**0.48** ^*^	0.40	0.47^*^
*setA*	0.39	**0.64** ^*^	0.60^*A^	0.52^*^	0.55^*^	**0.61** ^*^
*setB*	0.50^*^	0.67^*^	**0.69** ^*A^	0.61^*^	0.63^*^	**0.72** ^*AB^
*setC*	0.56^*^	0.70^*^	**0.76** ^*AB^	0.63^*^	0.65^*^	**0.74** ^*AB^

Correlation (*r*) values of breeding values predicted by OmicKriging or ElasticNet software vs. observed breeding values of families in batch three (N  =  22), using a model trained on batches one and two (N  =  56). Superscript * indicates a correlation p-value less than 0.05, superscript A indicates a combined model ANOVA fit significantly better than SNPs alone, and superscript B indicates a combined model ANOVA fit significantly better than Transcripts alone. Bold values highlight the strongest correlation within each software environment.

Using EN and applying removal of correlated features from SNPs (setB), resulted in a significant correlation (P  =  2.78e-3) (Table 3). Similar to what was observed when predicting both batches two and three, setA predictions with transcripts increased accuracy in both statistical methods and applying the removal of correlated features (setB) further improved predictions. (EN: P  =  1.57e-3 and OK: P  =  6.56e-4). The highest correlation within setB was observed using EN with the combination of transcripts and SNPs (P  =  1.82e-4) (Table 3). The ANOVA model showed combining transcripts and SNPs was better in EN than using SNPs alone (P  =  2.53e-2) and transcripts alone (P  =  4.52e-2), but it was only significantly better than SNPs (P  =  8.87e-3) when using OmicKriging.

**Fig 5:**
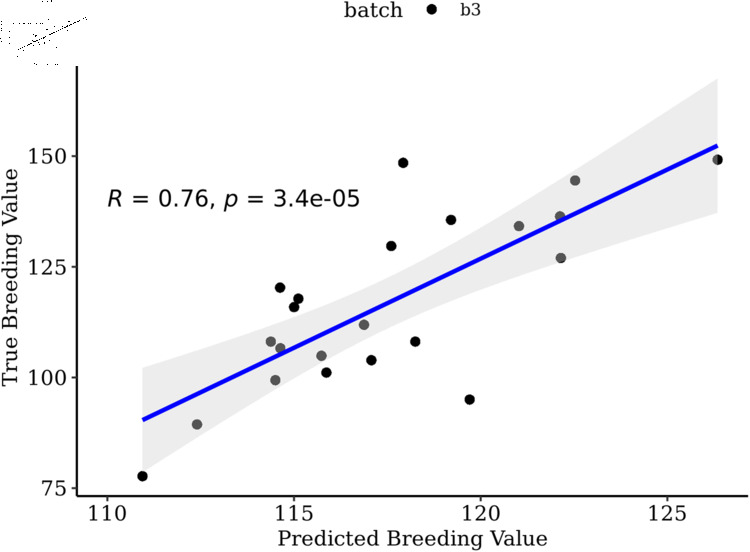
Predicted versus observed breeding values for batch three. Predicted versus true breeding values of batch three (N  =  22) families using setC combined predictions with OmicKriging. Training set included batch one and batch two (N  =  56).

ANOVA tests of association of the 5,174 SNPs genotypes with training population breeding values resulted in keeping 295 SNPs, and selection of transcripts correlated greater than *r*  =  0.05 with phenotypes retained 631 transcripts. Similar to the previous filters, combining the two data types performed the best in OmicKriging (P  =  3.42e-5) and EN (P  =  8.89e-5) (Table 3). The highest correlation when predicting batch three was using OmicKriging with the combined transcript and SNP predictions (Fig 5). The ANOVA comparison showed the combined SNP/transcript predictions from OmicKriging were significantly better than either transcripts (P  =  4.61e-2) or SNPs (P  =  2.43e-3) alone, and the same was true for EN SNPs (P  =  2.21e-3) or transcripts (P  =  3.33e-2) alone.

### Feature enrichment analysis

Feature-enrichment analysis of putative Arabidopsis homologues of the pine transcripts in setB and the three different versions setC (from training on batch one alone, batch one and two, or batch one and three) using gProfiler GOSt [54] showed enrichment in three or four of the transcript sets for some very general Gene Ontology terms (S1 File). These include “catalytic activity” (GO:0003824) in Molecular Functions, “response to stimulus” (GO:0050896) in Biological Processes, and “extracellular region” (GO:0005576) in Cellular Components. SetB transcripts were also significantly enriched in several more specific GO terms, some of which were also significantly enriched in one or more of the SetC transcript groups. These include Molecular Function terms “glucosyltransferase activity” (GO:0046527) and “UDP-glycosyltransferase activity” (GO:0008194), which are both related to carbohydrate polymer synthesis and could be connected to cell-wall formation in actively-growing seedlings.

Additional significantly-enriched Molecular Function terms are likely to be related to signaling cascades, including “protein serine kinase activity” (GO:0106310) and “protein serine/threonine kinase activity” (GO:0004674). These terms are still sufficiently general to make it difficult to ascribe specific functional roles for these pathways in the pine seedlings. It is interesting, however, that the same terms were significantly enriched in three or four of the sets of pine transcripts tested. This is consistent with the hypothesis that the filtering criteria used to create setB and the three versions of setC are actually working to remove background noise and enrich the signal of transcripts whose gene expression levels are stably correlated with breeding value for stem volume at age six years.

## Discussion

Genomic selection can potentially impact breeding of loblolly pine and other conifers by reducing the length of a given breeding cycle and increasing selection intensity or genetic gain made from a cycle [56]. In order to meet that potential, a few hurdles must be overcome, including the large genome size, significant heterogeneity, and low amount of linkage disequilibrium typical of conifers [57]. GS has been reported to work best within conifer populations that share high levels (0.12 to 0.5) of relatedness, equivalent to half-sib progeny (averaging 0.125) or full-sib progeny (averaging 0.5) or parent-offspring (0.5) relationships [28,29,58,59]. The cost-effectiveness of GS for different conifer species is likely to depend in part on the number and size of different training populations that must be genotyped in order to enable accurate prediction on a useful population of selection candidates [60].

In this study, we proposed the idea of enabling transcriptomic selection through the collection of RNA-sequencing data, and tested the concept utilizing three batches of seedlings produced from loblolly pine families spanning a range of breeding values for stem volume productivity. RNA-sequencing allows the collection of data on gene expression patterns and sequence variation within a single experiment, in contrast to array-based experiments which can provide either gene expression levels or SNP genotypes from a single assay [61]. One novel aspect of transcriptomic selection carried out in this experiment was aligning reads to a transcriptome reference rather than the genome reference and averaging the genotypes across biological replicates. Analyzing the transcriptomic data in this way allows us to average gene expression levels across members of multi-gene families represented by a single reference transcript, while potentially removing some environmental differences in gene expression.

When evaluating gene expression reproducibility, biological replicate transcript information showed a high degree of similarity. These results agree with previous studies showing the high reproducibility of expression estimates [62,63]. There was also a high similarity among replicates across batches, which provides confidence in reproducing certain expression levels across years and experiments if this method were used in the future. While family-mean expression levels were similar among families, the SNP relationship matrix showed minimal relationships among the families sequenced. This observation of high similarity of gene expression levels among families suggests that many genes may be expressed in the seedling stage as part of development independent of any family influence [64], and relatively few genes are expressed in family-specific patterns that may be correlated with mature patterns of gene expression associated with phenotypes. In keeping with this hypothesis, the highest predictive accuracies were generally obtained in this study with setC SNPs and transcripts, which are selected based on a low threshold level of association (*α*  <  0.05, uncorrected for multiple testing) with the breeding values used as phenotypes in the training population. We emphasize that these selected SNPs and transcripts detected in juvenile seedlings need not have any causal role in determining mature phenotypes to be useful in prediction; they only need to have a reproducible association with sequence variants or gene expression patterns that do have a causal role in determining phenotypes.

The lack of close relationships observed in the SNP-based relationship matrix makes this study unusual compared to most other genomic prediction experiments. Most previous omic prediction studies within row crops have relied on known pedigree relationships among the individuals sequenced [65]. Additionally, most forest tree genomic predictions have been evaluated using relatively close relationships between training and test populations. [66,67]. A recent assessment of prediction ability within a population of clonally replicated loblolly pine saw close to a 50% reduction in prediction ability upon removing full-sib relatives from the training population [28]. Based on the evidence that both gene expression and phenotype are heritable, it is reasonable to propose transcriptomics has some value in understanding differences in phenotypes among individuals [68,69]. The ability of this transcriptomic approach to achieve useful predictive accuracies across training and prediction populations that do not share known coancestry could make it particularly well-suited to the shallow pedigrees and high diversity characteristic of most forest tree breeding programs.

When training with a single batch, both the OmicKriging and EN models displayed relatively similar accuracies when using a single data type (SNPs or transcripts) in combination with a specific filter type. This observation tends to agree with previous genomic selection studies assessing ridge regression and elastic net [70,71], which concluded that the shrinkage of estimates does not significantly impact prediction accuracy when evaluating complex polygenic traits. The choice of statistical model (OmicKriging or ElasticNet) was less important than the choice of which predictor variables (SNPs or transcripts) were used in the modeling. Prediction with SNPs or transcripts that showed variation among families and were not highly correlated with one another seemed to have the largest impact, compared to using all data of either SNPs or transcripts or both. This observation makes sense given that EN has the potential to either include or remove a set of correlated variables [72].

Collinearity of the variables used in the correlation matrix could skew assumed relationships among individuals [73]. Surprisingly, in both OmicKriging and EN, the combination of SNPs and transcripts outperformed using either alone, suggesting that there may be different types of information from the two contributing to the prediction accuracy. Similar findings have been reported in maize, where prediction of dry matter yield and all other traits evaluated saw a slight boost in prediction accuracies when combining genotype and transcript information to predict hybrid phenotype [65].

It might be expected that combining batches to increase the size of the training population should provide higher prediction accuracies on the left-out test population used for prediction. We did not test for significant differences among the predictive accuracies between training only on batch one (N  =  45) and predicting batches two and three (Table 1), relative to training on batch one and two (N  =  56) to predict batch three (Table 3), but the overall trend of increasing predictive accuracy with increasing levels of filtering, and the magnitude of the best correlations, were quite similar. The largest training population (N  =  67), derivced from training on batches one and three and prediction of batch two families, gave a dramatically different outcome (Table 2). Using all the SNP genotype data, without filtering for variation among families or association with phenotype, came close to the level of accuracy observed with setC-filtered SNPs plus transcripts in Table 2, Table 3. The correlations of observed phenotypes for batch two with predicted phenotypes based on transcript levels were not significant at any level of filtration, nor were predictions based on both SNPs and transcripts better than predictions based on SNPs alone. The reason for the observed poor prediction accuracy of batch two using models trained on batch one and three is unknown. The fact that batches one and two were sequenced on a different instrument by a different sequencing facility than batch three may be an important factor, and the fact that the batch two prediction population is only 11 families may also be a factor.

One testable hypothesis is that the difference in sequencing technology between batches one and three confounds the filtering process, so that the SNPs selected after filtering give models with lower predictive power than using all SNPs. This was tested by applying the filtering steps separately to the batch one and batch three training populations, then using only SNPs that passed the filter in both independent populations, but this did not improve the accuracy of the predictions (data not shown). This hypothesis also does not account for the observation that the accuracy of the unfiltered SNP model in Table 2 is higher than the comparable model in Tables 1 and 3. An alternative hypothesis, which cannot be tested without additional sequencing experiments, is that the outcomes shown in Table 2 are due to sampling effects of families, because batch two contains only 11 families not present in batch one or three. It may be that by chance those 11 families are different in some way from the families in batch three, so that models trained with batch one alone predict batch two much more accurately than do models trained on batches one and three. The differences in sequencing technology between batches one and two and batch three may also play a role. The SNP relationship matrix shows a discernable differentiation between batch one/two and batch three using the filtered set of 214,449 SNPs, suggesting that these technical differences had some effect on the detection and genotyping of SNP loci, but this differentiation was removed by filtering out SNPs that show an association of genotype with batch in a single-factor ANOVA model. Further experiments will be required to determine the relative contribution of any of the above factors.

In practical terms, the results reported in Tables 1, 2, and 3 suggest some specific considerations for the design of future experiments on transcriptomic prediction in forest trees. One recommendation is to include a set of control families with a range of known breeding values in every batch of samples submitted for RNAseq library construction and sequencing. This would provide a basis for modeling batch effects and accounting for them in the selection of SNPs or transcripts for predictive modeling. A second recommendation is to ensure that every batch of samples submitted for predictive modeling contains at least 30 families, so the sample size for correlation analysis is large enough to avoid sampling variation and there is greater power to detect significant relationships between predicted and observed breeding values in the prediction population. A third recommendation might be to try to avoid changing sequencing platforms, but this is not likely to be a realistic possibility—the technology will change, and so designing experiments to be robust to batch effects will be important. One advantage of the rapidly changing technology has been that the costs of high-throughput sequencing have fallen dramatically over the past two decades, and are likely to continue dropping. These reduced costs will help make larger experiments that include multiple control families more cost-effective for applied breeding programs.

Genomic selection is generally applied as a form of forward selection, in which every offspring is individually-genotyped, and then those individual genotypes are analyzed, with or without phenotypes, to decide which individual to select for further breeding. Our transcriptomic selection approach is different; instead of genotyping individual offspring separately, we sequence them as a pool. The unit of selection is not a seedling from the genotyped set but the parent of the genotyped seedlings. The predictive accuracies reported here suggest this method may potentially be included as a form of very early selection, as it applies to the current loblolly pine breeding approach. In principle, both genomic and transcriptomic methods could be used together in the same breeding program by first using individual genotyping and genomic prediction to identify forward selection candidates. Those candidates could then be top-grafted and allowed to open-pollinate the first crop of female strobili produced. Those OP cones would be collected as soon as three years after top-grafting, and RNA-seq experiments conducted on a sample of the seedlings to predict parental breeding value based on transcriptome data. The combination of estimated breeding values predicted from genomic data of individual candidates, and those predicted from transcriptomic data of OP progeny of those candidates, could then be used to make final selections among the grafted forward selection candidates. The remaining OP seedlings could also be planted in field trials to get additional phenotypic data to update the models used to train the genomic and transcriptomic selection models.

Further improvement could be possible in the application of transcriptomic prediction by leveraging megagametophytes instead of the seedlings themselves. The megagametophyte is the nutritive tissue of the conifer seed, analogous to the endosperm of angiosperm seeds, but unlike the endosperm, the megagametophyte is derived from the same haploid product of maternal meiosis as the embryo. Preliminary data from a single family suggests that RNA-seq with RNA from megagametophytes yields results very similar to those obtained with RNA from seedlings, and may work just as well as RNA from growing seedlings. The ability to utilize megagametophytes would hasten analysis by saving the time required for seed stratification and seedling growth. Additionally, it may reduce the potential for batch effects related to greenhouse growth conditions.

## Conclusion

Overall, the findings from this study have the potential to provide a significant impact on how loblolly pine breeding populations are managed. Transcriptomic selection within loblolly pine would allow for future selections to be screened earlier, and allow greater selection intensity on breeding populations while also potentially providing more gain to be made over time. The exploration of using other omic technologies, besides strictly genomic selection, should continue to be of interest to loblolly pine breeders and potentially used in conjunction with genomic selection to provide the best crop moving forward.

## Supporting information

S1File Supplementary materials. Details of the experimental designs used to assess effects of lane and adapter index on the RNA-seq results are presented in Supplementary Materials, along with details on the origins of the loblolly pine families used in the experiments, and meta analyses.
(ZIP)
